# Distal Nerve Transfer for Opponensplasty in the Setting of High Median Nerve Injury: A Case Series

**DOI:** 10.1155/aort/8481129

**Published:** 2025-02-06

**Authors:** Mohammadreza Emamhadi, Mohammad Haghani Dogahe, Amirreza Emamhadi

**Affiliations:** ^1^Department of Neurosurgery, Guilan University of Medical Sciences, Rasht, Iran; ^2^Brachial Plexus and Peripheral Nerve Injury Center, Guilan University of Medical Sciences, Rasht, Iran; ^3^Guilan Road Trauma Research Center, Guilan University of Medical Sciences, Rasht, Guilan, Iran

**Keywords:** high median nerve injury, nerve transfer, neurotization, opponensplasty, peripheral nerve injury

## Abstract

**Background:** High median nerve injury leads to an absence of thumb opposition and irreversible thenar atrophy. Currently, distal nerve transfer is a new option for opponensplasty. The superiority of nerve transfer over traditional tendon transfer is that in nerve transfer, all thenar muscles may be reinnervated and so thumb functions are fully achieved, while in tendon transfer, the goal is to reanimate the function of abductor pollicis brevis (APB). This study aims to describe the results of opponensplasty using distal nerve transfer.

**Materials and Methods:** This article analyses the results of opponensplasty using the transfer of abductor digiti minimi (ADM) branch of the ulnar nerve to the recurrent branch of the median nerve. Clinical outcomes were assessed by objectively evaluating APB strength, degree of thumb opposition, and thenar muscle bulk. APB strength and degree of thumb opposition measured by Medical Research Council (MRC) and Kapandji scoring systems, respectively.

**Results:** From 2016 to 2019, six patients with a mean age of 29.5 years (five males and one female) with high median nerve injury were considered for opponensplasty using nerve transfer. Clinical improvement, including APB strength regaining and thumb opposition, was achieved in all patients. Moreover, recovery of thenar atrophy was observed in five patients.

**Conclusion:** In high median-nerve injury, early reconstructive intervention can prevent the thenar muscle atrophy and leads to prompt reinnervation and complete restoration of thenar function. ADM branch of the ulnar nerve is a superior donor for this purpose.

## 1. Introduction

Thumb opposition, consisting of palmar abduction, flexion, and pronation, is a unique feature in humans [1]. Having opposable thumbs enables humans to grasp things more efficiently, touch any point with fingers, and eat with one hand [2].

The median nerve plays a vital role in thumb opposition by innervating abductor pollicis brevis (APB) and other thenar muscles such as opponens pollicis and flexor pollicis brevis [3]. Median nerve injury above the elbow (high median nerve injury) is a rare condition that causes disability in thumb function because early exploration and repair do not ensure satisfactory results and prolonged reinnervation time will lead to irreversible thenar atrophy [4, 5].

In the setting of thenar atrophy, opponensplasty was first described by Steindler. Traditionally, a transfer of dispensable tendons to restore thumb opposition and restore tip and key pinch in median nerve palsy were the final approaches for these patients [6]. Despite the efficacy of tendon transfer for opponensplasty, new studies have shown that nerve transfer could restore thenar muscles and achieve satisfactory results in the early stages of injury. Compared with tendon transfer, which can only reconstruct one thumb function and is usually used to reconstruct thumb abduction, nerve transfer reinnervates all thenar muscles. As a result, nerve transfer can reconstruct at least three thumb functions, though it requires early-stage surgery with a short interval between injury and surgery to prevent irreversible atrophy. For that reason, if the patient is developing progressive atrophy following initial repair, it is crucial to consider nerve transfer at the earliest opportunity before motor endplate degeneration [7]. Supporting this idea, evolutions in distal nerve transfers have provided a new opportunity to minimize the drawbacks of direct nerve repairs in peripheral nerve injuries [5].

In this study, the authors have reported the results of early opponensplasty using nerve transfer in six patients with high median nerve injuries.

## 2. Methods and Methods

All patients with high median nerve injury who underwent opponensplasty were identified by reviewing surgery case logs between 2016 and 2019 of the senior author of the article. Primary repair has been performed after injury. The patients were referred for reconstructive surgery by nerve transfer a few months after primary nerve repair. All patients had some degree of thenar atrophy. Reconstructive surgery was performed by nerve transferring from the Abductor Digiti Minimi (ADM) branch of the ulnar nerve to the recurrent branch of the median nerve (RMN). The study was approved by our ethics committee review board and all patients confirmed informed consent before recording data.

**Figure 1:**
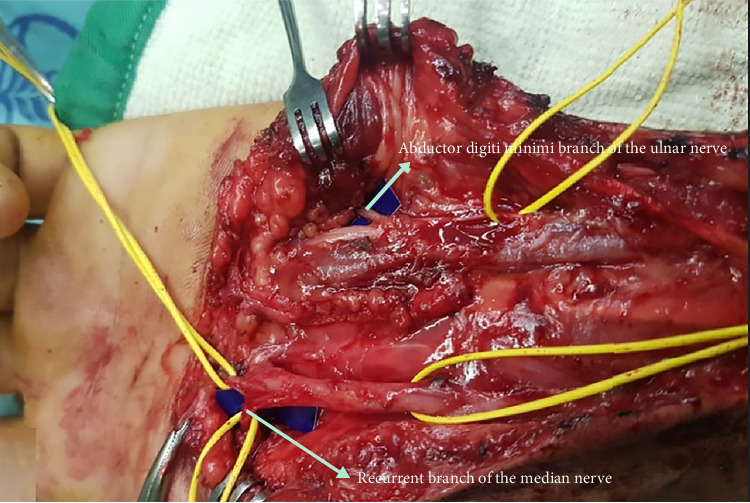
Opponensplasty using abductor digiti minimi branch of ulnar nerve transfer to the recurrent branch of median nerve.

Electrodiagnostic test was done prior to the surgery to confirm integrity of donor (ulnar) nerve and to show the target (thenar) muscles denervation. There was no EMG evidence of recovery after primary nerve repair, and denervation of the thenar muscles was confirmed based on serial EMG findings. The operation was performed under general anesthesia in the supine position. A curvilinear incision between the hypothenar and the thenar origin was made. After opening the Guyon canal, the ulnar nerve was exposed. The ADM branch of ulnar nerve was identified and its function as a donor was evaluated using electrical stimulation. Subsequently, to expose the median nerve, the medial side of the flexor retinaculum was divided and the thenar branch of the median nerve was identified as recipient nerve. The thenar branch was divided a few centimeters proximally. The ADM branch was cut as distal as possible and transposed toward the thenar branch by end-to-end transfer using 9-0 monofilament sutures (Figure 1). All surgeries were performed without any complications. One month splint following the procedure and then continuous physiotherapy was considered for the patients. The hand was placed in splint in a “resting position.” We believed that the denervated muscles should rest for one month and subsequently physiotherapy should be started for them.

Three types of current stimulations could be used for electrical stimulation: (1) galvanic stimulation uses a direct current, (2) pulsating direct current and (3) alternative current. Interrupted direct current (IDC) was used in our patients because direct current creates an electrical field over the affected area, changing blood flow to promote healing and stimulate any denervated muscle or lacking proper nerve function.

One of the most important parts of rehabilitation is cognitive training and motor re-education. In this situation, while the patient attempts to abduct the small finger, thumb abduction was performed passively. The scientific basis for cognitive training is that the motor system is part of a cognitive network that includes various psychological activities. Neural pathways are activated during cognitive training and actual performance of a task and brain changes resulting from cognitive training for a specific motor task mimic those observed after physical practice of the same skill.

Evaluation of the pre- and postoperative thumb function was assessed by clinical grade of APB strength, degree of thumb opposition, and thenar muscle bulk in follow-up visits and the aforementioned gradings for thumb function were recorded by the senior author of the article.

**Figure 2:**
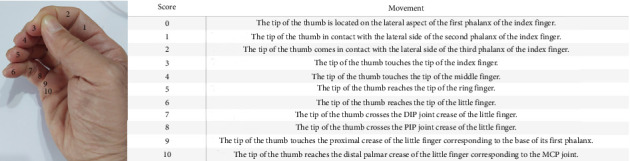
Kapandji scoring. The first three stages (stages 0, 1, and 2) along the path describe a terminolateral pinch and the second part (stages 3–6) of this path describes the course of a tip-to-tip pinch. In the last part of the path (stages 7–10), the tip of the thumb runs on the volar aspect of the little finger to extreme opposition. A score of 0 indicates no opposition, and a score of 10 indicates maximal opposition.

**Figure 3:**
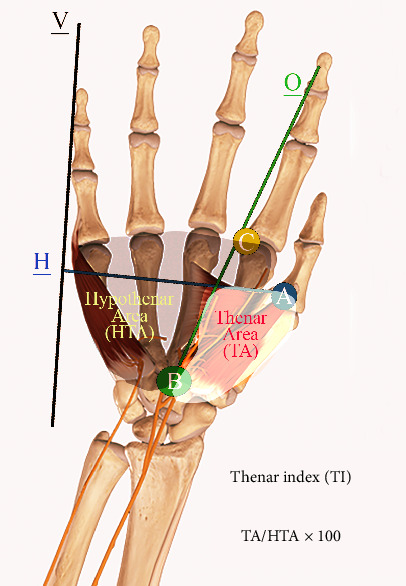
A static hand imprint showing key landmark points and lines on the contact area. (Above, left) Point A: located at the junction of the proximal metacarpophalangeal joint crease of the thumb and the palm in the first web space; Point B: where the skin line formed by the confluence of the thenar and hypothenar areas meets the distal wrist crease; Point C: at the metacarpal head of the index finger, marking the second ray. (Above, right) Line H: a horizontal line from point A; Line O: an oblique line extending from point B to point C; Line V: a vertical line used solely to align the hypothenar side vertically, not affecting the isolation of the thenar area. (Below, left) the thenar area (TA) is isolated, with outlines of the thenar area (TA) and the remaining hand area (HTA) drawn. (Below, right) these outlines are transferred to a new image for area calculation, with the thenar index formula included. A 2 mm upward shift of line H indicating complete recovery and full functional restoration; a shift between 2 mm upward and 2 mm downward of line H, suggesting mild atrophy with slight muscle size reduction; moderate atrophy, characterized by noticeable muscle wasting but retained ability for thumb opposition, accompanied by a 2-degree tilt of line O toward the thenar area; and severe atrophy, marked by significant muscle loss, a 2-degree tilt of line O toward the hypothenar area, and an inability to perform thumb opposition effectively [12].

APB strength was scored from 0 to 5, in accordance with the British Medical Research Council (MRC) rating scale [8]. The degree of the thumb position was measured according to Kapandji tip opposition which is classified based on where the tip of the thumb can touch the other fingers (Figure 2) [9]. Thenar muscle atrophy was divided into four grades, from 0 to 3 [10]. Grade 0 denoted a complete recovery. Grade 1 (mild) indicated a slight decrease in muscle size and mild weakness compared with the unaffected side, while grade 2 (moderate) referred to noticeable muscle wasting but still retaining the ability for thumb opposition. Grade 3 (severe) referred to significant muscle atrophy and was unable to perform thumb opposition effectively [11]. Although the aforementioned classification by Mackinnon et al. is an objective assessment, the thenar index provides a more accurate measurement, as depicted in Figure 3 [12].

## 3. Results

A total of six patients (one woman and five men) were surgically treated for high median nerve injuries. Two out of six patients had a knife injury, one glass injury, one car accident, and one stone cutting machine. The median nerve was injured at the level of arm in five patients, and in one patient, the injury was at the elbow level.  Table 1 summarizes demographics, clinical data, and outcomes of early opponensplasty using nerve transfer in the patients.

The average age at the time of surgery was 29.5 years. Primary median nerve repair was performed in all patients except for 2 cases who required autograft.

We performed end-to-end nerve transfer of the motor branch of the ADM to the thenar branch of the median nerve to improve thumb motion and pinch strength and in order to prevent more thenar atrophy. The mean interval between injury and surgery in our cases was 3.6 months.

The patients were followed for 12–18 months (mean = 14.1 months). Following the surgery, clinical improvement in APB strength and Kapandji score was obtained in all patients. Three months after the nerve transfer surgery, EMG studies showed a decrease in fibrillations and the appearance of polyphasic waves along with motor unit action potentials (MUPs), indicating reinnervation in the thenar muscles. The preoperative APB strength was limited to M1. Following nerve transfer, postoperative APB strength improved to a mean of M3.8 (range: M3–M4), with five patients achieving M4 and one patient reaching M3, reflecting a substantial gain in thumb abduction strength (Video 1). The preoperative Kapandji score ranged from 0 to 2, reflecting limited thumb opposition function. Postoperative recovery of the Kapandji score reached a range of 6–10, with a mean increase from 1.2 preoperatively to 7.0 postoperatively. All patients achieved a Kapandji score of ≥ 6 at the final follow-up.

**Figure 4:**
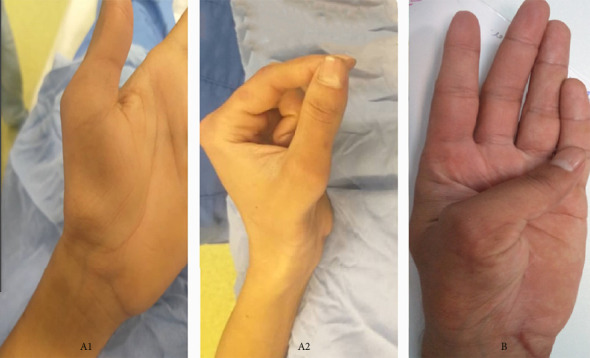
Comparison of thenar bulk and thumb opposition before and after the surgery at the last follow-up in one of the patients.

The thenar bulk was recovered in five patients.  Figure 4 illustrates the improvement of thenar bulk and thumb opposition at the last follow-up comparing preoperative severe thenar atrophy. Thenar muscle atrophy ranged from moderate to severe preoperatively. Postoperatively, five patients showed visible improvement in thenar bulk, with a mean reduction in atrophy score from 2 to 0.8, demonstrating significant restoration of muscle volume and reduced atrophy in most cases (Table 1).

In our study, no complications or deficits were observed in the postoperative period. According to our observations, we consistently identified at least one branch connected to the ADM muscle. However, it is important to note that the ADM muscle occasionally receives multiple nerve supplies.

## 4. Discussion

The management of thenar atrophy following high median nerve injuries is challenging and clinical outcomes are variable. In this study, the authors present the results of early distal nerve transfer for restoration of thenar muscles in six patients with high median injury.

In the setting of high median nerve injury, reconstructive surgery is mainly aimed at restoring the thumb opposition. Primary exploration and repair are indicated in penetrating nerve injuries which provide the best chance for recovery of proximal muscles and restoration of sensation.

The potential for complete recovery of the median nerve after repair, especially in cases of high median nerve injury, is influenced by several critical factors. Studies indicate that timing of the repair, type of nerve injury, patient health factors, and the techniques used for nerve repair are all essential in achieving optimal outcomes. According to Emamhadi et al., early intervention increases the likelihood of successful motor and sensory recovery by minimizing axonal degeneration and preserving motor endplates, which are crucial for reinnervation. In addition, injuries with lesions “in continuity' repaired with direct suture yield better results than those requiring grafting [13].

When the thenar muscle is preserved, muscle bulk and responsiveness to regenerating axons are maintained, which enhances the chances for functional recovery. However, even with early repair, complete recovery is challenging due to factors such as axonal misdirection, scarring, and the slow regeneration rate of approximately 1-2 mm per day [14]. These limitations are especially relevant for high median nerve injuries, where axons must regenerate over long distances to reach target muscles, making full functional recovery less likely.

Ronchi et al. emphasized that adjunct therapies, such as nerve guidance conduits, electrical stimulation, and stem cell applications, can improve reinnervation outcomes by supporting axonal growth and minimizing scarring. However, even with these interventions, achieving complete recovery can be hindered by regeneration barriers, particularly in more proximal injuries [15].

Given these limitations, the use of nerve transfer offers an effective alternative, as applied in this study. This technique can bypass some of the regenerative distance, enabling reliable motor control, especially for thumb opposition. Therefore, while primary repair may hold some potential for complete recovery in ideal scenarios, nerve transfers provide a more consistent and effective means of restoring function in complex or delayed cases [4, 7].

Since thenar eminence muscles mainly control thumb opposition, traditional reconstructive approaches were based on tendon transfers [16]. Steindler et al. originally described opponensplasty in median nerve palsy to restore thumb opposition and top and key pinch using tendon transfer. Since that time, various tendon, muscle, and nerve transfers have been used for this purpose [6, 17–19].

Tendon or muscle transfer may be the last option for opponensplasty. The main unfavorable concerns about tendon transfers are prolonged immobilization of small joints which leads to contracture, requirement of extensive hand therapy, abnormal biomechanics, poor cosmesis, and failure [20]. ADM muscle transfer can well restore abduction, but rotation and flexion are somewhat less well achieved [21].

Studies have shown that both tendon transfer and nerve transfer techniques have their advantages and disadvantages. For example, tendon transfer is a well-established technique with proven durability and reliability. It has been shown to provide good functional results, even in patients with severe nerve injuries. Anderson and colleagues conducted a study to compare the results of the utilization of the flexor digitorum superficialis of the ring finger and the extensor indices (EIs), which revealed that EI opponensplasty was more effective in flexible hands, while FDS opponensplasty was more appropriate for less adaptable hands [22].

Current trials have shown that nerve transfer could be effectively used in high median nerve injuries [7]. Distal nerve transfer for opponensplasty yields more rapid recovery of thumb function and motor strength, limited antagonistic cocontraction, reinnervation of all thenar muscles, and reversal of atrophy. Notably, in cases in which nerve transfer fails, tendon transfer is still available [20, 23]. Nerve transfer to the thenar muscles should be done before motor endplate degeneration.

In the high median nerve injuries, one of the main challenging is that few nerves are available as a donor for reanimation of thenar musculature by reinnervating RMN as a recipient. The muscular branch of the ulnar nerve to the third lumbrical muscle (3rdLn) and flexor digiti minimi brevis (FDMBn) appear to be desirable as suitable donors since they provide the shortest time for the donor fibers to reinnervate RMN. Moreover, the synergistic activation of fifth finger palmar flexion could facilitate relearning of thumb opposition and palmar abduction [20]. Nevertheless, the diameters of these two branches are substantially smaller than the RMN.

Schultz and Aiache first explained the surgical technique of transferring the 3rdLn transfer to RMN in a 22-year-old man who sustained a knife wound resulting. Eleven weeks after nerve transfer, thumb abduction and pinch functions recovered without thenar atrophy [23].

Ozcelik et al. reported very high satisfaction in two patients who underwent opponensplasty by end-to-end transferring of the first palmar interosseous muscle of the ulnar nerve to the thenar branch of the median nerve [7].

Bertelli et al. transferred the motor branch of the ADM for thenar muscle reinnervation in eleven patients with high median nerve injury and achieved satisfactory results [5]. The results of this study support the efficacy of this nerve transfer.

Cadaveric studies have shown that the extensor indicis proprius branch of the posterior interosseous nerve, the ulnar motor branch to the flexor digiti minimi brevis, and the ulnar motor branch to the third lumbrical may be used as a donor for reanimation of thenar muscles but the clinical efficacy of these donors has not been shown yet [20].

The best outcome of nerve transfer would be achieved in the early stage of the injury. Prominent atrophy of the thenar and forearm muscle masses will occur between twenty-six and thirty-four days after injury. Therefore, motor nerve transfer should be done within the first month following surgery to prevent permanent thenar atrophy [4].

We decided to conduct an end-to-end nerve transfer because we strongly believe that it offers superior functional outcomes and quicker recovery compared to end-to-side nerve transfer. This preference stems from the fact that end-to-end nerve transfer preserves the natural pathway of the nerve, enabling the patient to achieve more accurate control and refine muscle movements. Moreover, end-to-end nerve transfer is a widely recognized and extensively utilized technique in the realm of nerve surgery

Although it is true that some patients may not be suitable candidates for nerve transfer procedures; we believe that the only contraindication of nerve transfer in the setting of high median nerve injuries is a coincidence of median and ulnar nerve injuries. Although there may be some relative contraindications in some instances, such as advanced age, coexisting medical conditions, or severe comorbidities, the final decision as to whether or not a patient is a suitable candidate for nerve transfer surgery should be based on a comprehensive evaluation of their condition.

Finally, it should be noted that while case series studies can provide useful insights into the outcomes of a surgical procedure, they have limitations that need to be carefully considered when interpreting the results, such as small sample size, selection bias, lack of comparison group, retrospective nature, and follow-up time. Further studies with larger sample sizes, comparison groups, and longer follow-up times may provide more robust and generalizable results.

## 5. Conclusion

The clinical improvement observed in terms of thumb opposition and thenar bulk in our cases confirmed the effectiveness of nerve transfer for opponensplasty.

## Figures and Tables

**Table 1:** Patient demographics, clinical data, and outcomes of opponensplasty with nerve transfer.

Case no.	Age/gender	Mechanism of trauma	Level of injury	Primary median nerve repair	Injury to surgery interval (months)	Follow-up (months)	Pre vs. postoperative thenar atrophy (grade)	Postoperative APB strength (MRC)	Kapandji score (Pre vs. postoperative)
1	32/M	Glass injury	Elbow	Direct	3	12	2 to 1	4	2 to 8
2	22/F	Knife injury	Upper arm	Direct	4	18	2 to 0	4	2 to 6
3	19/M	Car accident	Upper arm	Direct	2	15	1 to 1	4	1 to 6
4	26/M	Gunshot injury	Upper arm	Graft (3 cm)	6	12	3 to 1	4	0 to 6
5	45/M	Stone cutting machine	Mid arm	Graft (5 cm)	4	12	2 to 1	3	1 to 6
6	33/M	Knife injury	Upper arm	Direct	3	16	2 to 1	4	1 to 10

*Note:* The results of opponensplasty with nerve transfer; gradings which is used to compare pre- and postoperative thumb function are defined in the method section. Thenar muscle atrophy was assessed using a grading system as none (0), mild (1), moderate (2), or severe (3) based on the bulk of the abductor pollicis brevis muscle [10].

Abbreviation: MRC, Medical Research Council.

## Data Availability

The data used to support the findings of this study are available from the corresponding author upon reasonable request.

## Supporting Information

Video 1 (Supporting). Watch the remarkable progress: From nerve injury to functional recovery. Witness the journey of a patient postreconstructive surgery, demonstrating improved hand function and increased thenar bulk.
